# Cross-linking reactions in Langmuir monolayers of specially designed aminolipids – a toolbox for the customized production of amphiphilic nanosheets†

**DOI:** 10.1039/d3na00244f

**Published:** 2023-08-09

**Authors:** Cristina Stefaniu, Christian Wölk, Victoria M. Latza, Andrei Chumakov, Gerald Brezesinski, Emanuel Schneck

**Affiliations:** a Departments of Biomaterials and Biomolecular Systems, Max Planck Institute of Colloids and Interfaces Am Mühlenberg 1 14476 Potsdam Germany; b Pharmaceutical Technology, Faculty of Medicine, University of Leipzig Eilenburger Str. 15a 04317 Leipzig Germany christian.woelk@medizin.uni-leipzig.de; c European Synchrotron Radiation Facility 71, avenue des Martyrs, CS 40220 38043 Grenoble Cedex 9 France; d Department of Physics, TU Darmstadt Hochschulstr. 8 64289 Darmstadt Germany emanuel.schneck@pkm.tu-darmstadt.de

## Abstract

Synthetic amino lipids, already known as highly efficient gene therapy tool, are used in a novel way to create cross-linked stable one-molecule-thin films envisioned for future (bio)-materials applications. The films are prepared as Langmuir monolayers at the air/water interface and cross-linked ‘*in situ*’ *via* dynamic imine chemistry. The cross-linking process and the film characteristics are monitored by various surface-sensitive techniques such as grazing incidence X-ray diffraction, X-ray reflectivity, and infrared reflection–absorption spectroscopy. After transfer onto carbon grids, the cross-linked films are investigated by transmission and scanning electron microscopy. The obtained micrographs display mechanically self-supported nanosheets with area dimensions over several micrometers and, thus, an undeniable visual proof of successful cross-linking. The cross-linking process at the air/water interface allows to obtain Janus-faced sheets with a hydrophobic side characterized by aliphatic alkyl chains and a hydrophilic side characterized by nucleophilic groups like amines, hydroxyl groups and imine.

## Abstract 2

Mechanically stable nanosheets have been achieved by cross-linking reactions at the air/liquid interface. The monolayers and reactions are characterized by surface-sensitive techniques, and transferred Janus-faced films are investigated by TEM/SEM.

## Graphical abstract

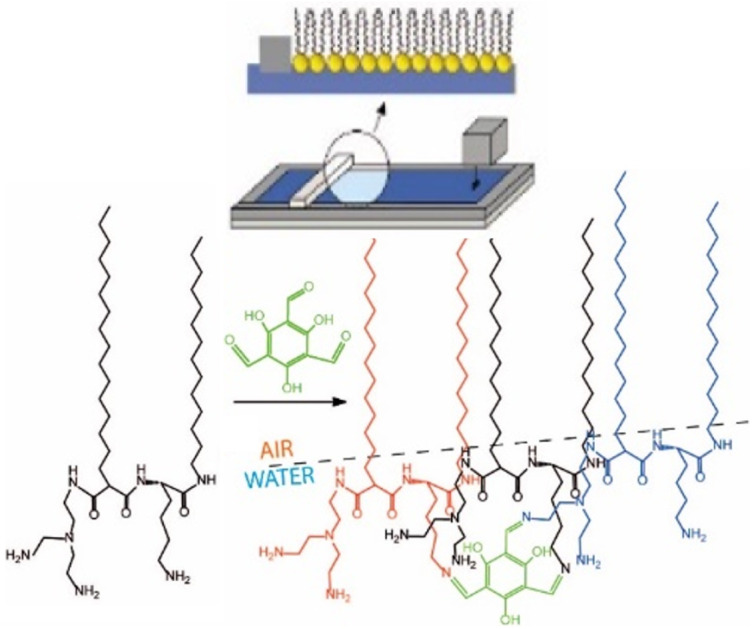

## Introduction

Two-dimensional (2D) nanosheets have been highly appreciated as investigation playground for biophysical fundamental research, where they have been accepted as cellular membrane models.^1^ In this context, specially-designed molecules containing polymerizable groups in the hydrophobic and/or hydrophilic parts of the molecules have been used to obtain stabilized biomembrane models *via* photo-polymerization in monolayers and in Langmuir–Blodgett films (LBF). In addition to their use in fundamental research, such polymer monolayers and LBF have been employed in modern engineering as calibrated insulating and active layers for metal–dielectric–metal and metal–dielectric–semiconductor systems and also as optical and electron beam resistors in high-resolution microlithography.^2^ The last years have witnessed growing interest in molecularly-thin 2D films generated with dynamic covalent chemistry at the air/water interface^3,4^ and 2D polymers (2DP) have been obtained as monomolecular covalent networks of periodically linked monomers. Such 2DPs are particularly interesting for surface and materials science due to their special properties which are sometimes different from their bulk analogues.^3,4^ In this context, coordinated nanosheets or covalent organic frameworks dominate the field of cross-linking reactions at the liquid/liquid or air/water interface.^5–9^ Also polymerization of Langmuir monolayers induced by UV irradiation^10,11^ or other chemical reactions^12^ were described. Chemical cross-linking of amphiphiles at air/water or liquid/liquid interfaces also appears to be a promising strategy to create soft interfaces with defined mechanical properties (such as dilation, shear, and bending moduli), transport properties through pores, and interaction characteristics with solutes and other interfaces, the latter being of relevance for colloidal stability. The synthesis of 2DPs directly at the air/water interface is of outstanding interest because monolayers are immediately produced and exfoliation is not required. Furthermore, large lateral sheet sizes can be obtained.^13^

Here, we report on the use of the so-called dynamic imine chemistry which is defined by the formation of a Schiff base (R1–HC=N–R2) between an aldehyde (R1–HC=O) and an amine (R2–NH_2_) group when applying basic conditions (pH > 9).^14,15^ Such Schiff base reactions have previously been used for the preparation of ordered 2D covalent organic frameworks (COFs),^16,17^ extended monolayer structures by on-surface approaches, and even sub-nanometer thin freestanding monolayer sheets at the air/water interface.^18–20^ However, those 2DPs have no amphiphilic properties. The polymerization of amphiphiles at the air/water interface is a strategy for the production of 2DPs with amphiphilic character. For instance, this was realized with amphiphiles containing hexayne structures.^10^ A UV-light-mediated carbonization of the hexayne structure has been used as 2D polymerization reaction to synthesize sheets of ≈2 nm thickness.

**Fig. 1:**
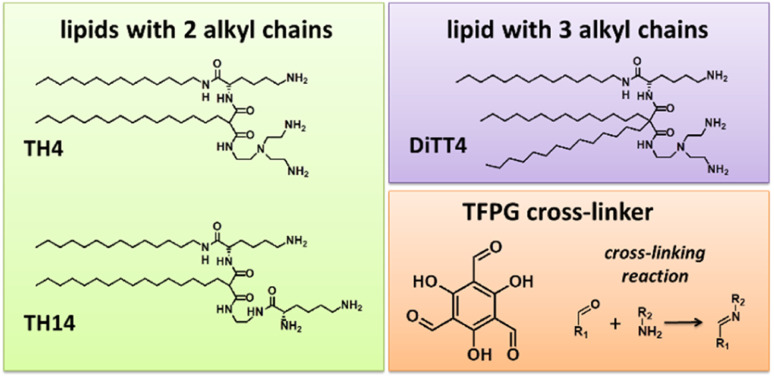
Structures of the aminolipids used to form Langmuir monolayers, which were subsequently cross-linked with the cross-linker (TFPG). The cross-linking reaction is schematically indicated. The reaction is based on the carbonyl activity of TFPG which forms imine bonds (Schiff base formation) with primary amino functions of the lipids.

The present work focusses on a versatile, light-free cross-linking reaction in Langmuir monolayers formed by specially designed synthetic aminolipids with the aim of obtaining mechanically stable, cross-linked amphiphilic nanosheets. The primary amino functions in the hydrophilic lipid head group allow the formation of imine functionalities after reaction with aldehydes dissolved in the subphase. In recent years, such amino-functionalized lipids have been synthesized and tested with the aim to find most suitable gene carriers for non-viral transfection.^21^ Our recently reported physical–chemical study revealed the ability of these aminolipids to form stable one-molecule-thin films at the air/water interface.^22^ For these films, the thicknesses of the hydrophilic and hydrophobic parts was controlled on an Angstrom level by the choice of the aminolipid *via* the interplay between molecular geometries and intermolecular interactions. In the present work, we provide a proof-of-concept by using different aminolipids (Fig. 1) to perform cross-linking reactions in the headgroup region with the above-mentioned dynamic imine chemistry. The lipids have three primary amino functions in the hydrophilic headgroup. This multi-valency allows the formation of a macroscopic network after the reaction with a multivalent aldehyde as cross-linker. Although all lipid species are trivalent primary amines, TH4 and DiTT4 have a comparable headgroup but different packing requirements due to the alkyl chains, while TH14 is characterized by a different headgroup structure. The imine condensation reaction was employed to cross-link the amino groups with a water-soluble tri-functional aldehyde, namely the 1,3,5-triformylphoroglucinol (TFPG, see Fig. 1), which was already successfully used for such reactions.^23–26^

The arrangement of the reactive moieties at the interface in a favourable position is crucial to succeed in a 2D polymerization to form Janus-faced amphipathic polymer sheets due to the forced orientation of the lipid monomers. This pre-organization can be controlled by changing the packing density of the starting monolayer and thus the mean distance between monomers monitored by area/pressure isotherms. Based on these results, we decided to perform the cross-linking reaction on structured Langmuir monolayers at high surface pressures, *e.g.* π = 30 mN m^−1^. The changes produced by the cross-linking reaction were monitored *in situ* with various surface-sensitive characterization techniques such as grazing incidence X-ray diffraction (GIXD), X-ray reflectivity (XRR), and infrared reflection–absorption spectroscopy (IRRAS). Transmission electron microscopy (TEM) and scanning electron microscopy (SEM) were then used subsequently for visualization of the films transferred from the air/water interfaces onto support grids.

## Results and discussion

Various surface-sensitive methods were used to follow the polymerization process *in situ*. However, not every analytical technique was applied to every lipid species because of limited availability of synchrotron-based methods and material limitations not allowing to repeat unsuccessful experiments. Nevertheless, the present work reveals the potential of each method and its limitations. Our study can be considered a proof-of-concept to provide clear evidence of the cross-linking process and to illustrate strategies using surface-sensitive techniques to monitor the polymerization reaction at the interface.

**Fig. 2:**
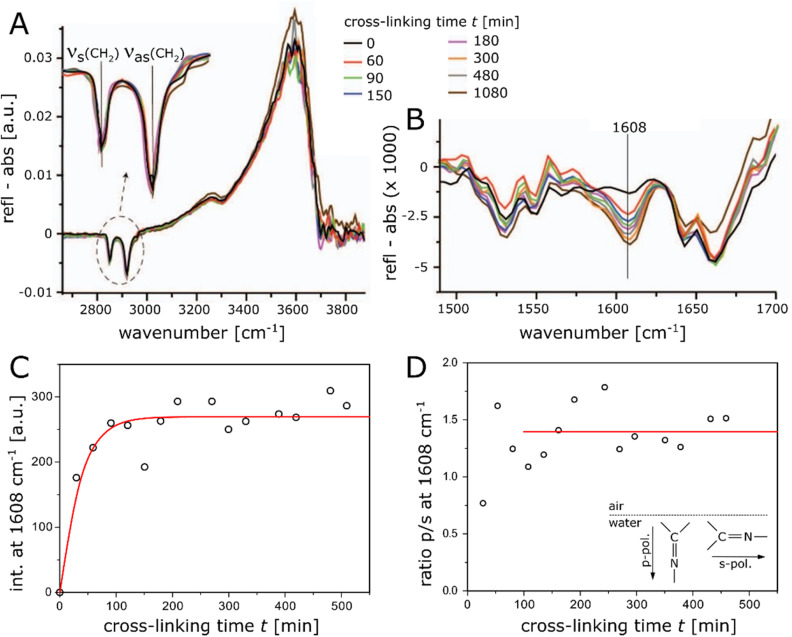
(A) IRRA spectra recorded during 18 hours of cross-linking time of TH14 Langmuir monolayers kept at a constant pressure of π = 30 mN m^−1^. (B) Spectra showing an increase in intensity of the characteristic imine –C=N-band at 1608 cm^−1^ as an indication of the progression of the cross-linking reaction. (C) Intensity of the newly formed imine –C=N-band at 1608 cm^−1^ as a function of the cross-linking time. The solid red line is an empirical fit that serves to guide the eye. (D) Evolution of the calculated dichroic ratio (*I*_p_/*I*_s_) of the imine –C=N-band at 1608 cm^−1^. The solid red line indicates the average value after saturation. Inset: schematic representation of the orientation of the imine groups in a plane perpendicular to the air/water interface.

IRRAS experiments are ideally suited to monitor the progression of the cross-linking reaction through the appearance/disappearance of signals of functional groups and band shifts, which are sensitive to structural changes in the lipid monolayers. Fig. 2 shows time-dependent changes in the IRRA spectra taken at an incidence angle of 40° on carbonate buffer subphases at 20 °C for TH14 Langmuir monolayers under the influence of the cross-linking reactant (carbonate buffer at pH 10, see Experimental for further details), which was injected into the aqueous subphase. TH14 is a trivalent amino lipid with a bulky headgroup dominating the area per molecule, resulting in a strong tilt of the alkyl chains. An intermolecular hydrogen bond lattice was reported.^22^ The monolayer was compressed to π = 30 mN m^−1^ and the surface pressure was kept constant during the reaction. Note, however, that pressure sensing with the Wilhelmy technique is not particularly suited for stiff monolayers (see ESI†). The intensities of the OH-band (a semi-quantitative measure of the effective layer thickness) do not change during the reaction (Fig. 2A). The positions of the CH_2_ stretching-bands (a measure of packing density and phase state of the chains) indicate an all-*trans* conformation of the chains at the beginning of the reaction. During the reaction, the positions do not change at all (asymmetric stretching vibration, *ν*_as_) or change only marginally to higher wavenumbers (better seen in the symmetric stretching vibration, *ν*_s_) and remain rather characteristic for liquid-ordered chains^27–29^ than for liquid-disordered (*gauche* conformation) chains. The progress of the cross-linking reaction was quantified by the intensity increase of the characteristic imine –C=N-band^3^ at 1608 cm^−1^ (Fig. 2B), the connective bond between lipid and cross-linker. The evolution of the intensity of this band (Fig. 2C) demonstrates that a plateau region is reached within approximately two hours reaction time, indicating the saturation of the polymerization process. The solid red line indicates an exponential saturation fit, *I*(*t*) = *I*_0_[1 − exp(−*t*/*τ*)], which yields the saturation time scale *τ* = (32 ± 7) min and serves as guide to the eye. Regarding the dichroic ratio *I*_p_/*I*_s_ of the imine band (Fig. 2D), a value significantly above unity (*I*_p_/*I*_s_ ≈ 1.4, averaged over time after saturation, see horizontal red line), suggests that the newly formed –C=N– bond has a near-perpendicular orientation to the air/water interface, as schematically illustrated in the inset of Fig. 2D. This fact allows assuming that the reaction with the cross-linker has a suprafacial orientation, meaning that the attached lipids are orientated on one side of a cross-linker molecule.

**Fig. 3:**
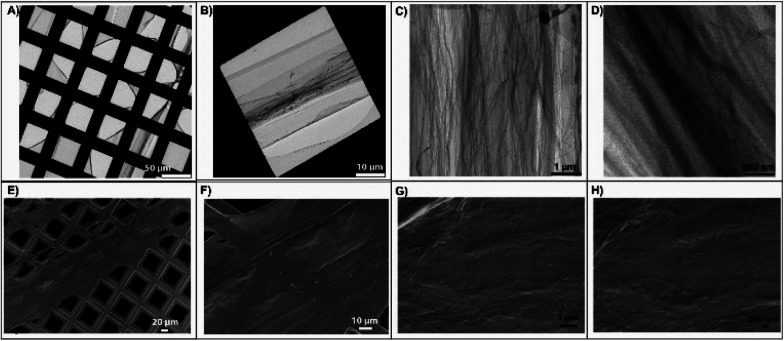
Transmission electron microscopy (TEM, panels (A–D)) images and scanning electron microscopy (SEM, panels (E–H)) images at various magnifications of TH14 cross-linked nanosheets after Langmuir–Schaefer transfer onto supporting grids. The images illustrate the existence of micrometer-large films, which can form wrinkles (better depicted in (C)) when over-compressed (close to the barrier region).

The IRRA spectra clearly indicate the formation of the imine bond but do not provide information about the formation of macroscopically cross-linked sheets. Therefore, the monolayers after reaction were manually transferred onto solid supports (Quantifoil® Holey Carbon support grids) and characterized by TEM and SEM. The images reveal free-standing cross-linked nanosheets that span the 40 × 40 μm^2^ sized holes of the grids (Fig. 3A and E). The TEM and SEM micrographs demonstrate that the films are characterized by both uniform and smooth surfaces and also by partially draped and wrinkled regions (Fig. 3B–D). The latter could be explained by an *in situ* over-compression of the Langmuir monolayer during the transfer from a region close to the compression barrier or by the rupture of the films due to local sample damage occurring especially during the TEM measurements.

**Fig. 4:**
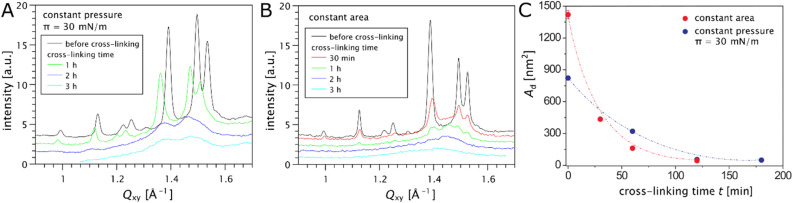
Changes of the diffraction patterns induced by the cross-linking reaction as obtained by GIXD measurements of DiTT4 monolayers. (A) Experiments performed at constant lateral pressure, π = 30 mN m^−1^. (B) Experiments performed at constant area. (C) Temporal evolution of the average area of crystalline domains *A*_d_ during the cross-linking reaction for both boundary conditions.

Synchrotron-based GIXD and XRR were performed to follow the cross-linking process in the lipid monolayers by observing changes in the in-plane and out-of-plane structures in the 2D layers. Interestingly, the surface pressure at the air/water interface was found to increase during the cross-linking reaction performed at constant surface area, resulting from an increase in the area requirement of the cross-linked film. Although pressure measurements are not so reliable when the monolayer gets cross-linked, they provide qualitative information on the monolayer's area requirement. GIXD was therefore performed both at constant surface pressure and at constant surface area. The results using a DiTT4 Langmuir monolayer on carbonate buffer at pH 10 are presented in Fig. 4. The trivalent aminolipid DiTT4 was selected for these experiments because its untreated Langmuir monolayers are characterized by a liquid-condensed (LC) phase with high structural order:^22^ a strong H-bond intermolecular network between the amine–amide headgroups and strong non-polar interactions between the three alkyl chains lead to a molecular superlattice. The three strong diffraction peaks above the horizon (*Q*_*z*_ > 0) in the wide-angle region (at high *Q*_*xy*_) reveal an oblique lattice structure of chains (*b* = 5.023 Å, *c* = 5.150 Å, *α* = 125.4°) with a chain tilt of *φ* ≈ 19° (see Tables S1 and S2 in ESI†). The additional Bragg peaks found in the mid-angle region (0.9 Å^−1^ < *Q*_*xy*_ > 1.3 Å^−1^) arise from the molecular superlattice (Table S3†). The supercell has an area of 126.5 Å^2^, is commensurate with the hydrocarbon chain lattice (*b*′ = 2*b*, *c*′ = 3*c*) and contains two DiTT4 molecules with an in-plane area per chain of *A*_*xy*_ = 21.1 Å^2^. The results obtained in the present work and in a previous study^22^ are in good agreement except of the average crystalline size determined from the peak widths in *Q*_*xy*_-direction (see ESI†), where the difference must be attributed to differences in the spreading and compression procedures. The full-width at half-maximum (FWHM) of the peaks in *Q*_*z*_-direction (Bragg rods) agrees well with the length of an extended C14 alkyl chain in all-*trans* conformation (see ESI†). Fig. 4A and B show that the progression of the cross-linking reaction leads to a decrease in the intensity of the diffraction peaks, their shift to lower *Q*_*xy*_ values (more pronounced in the constant pressure configuration), as well as their progressive broadening. The in-plane area of the molecules in the ordered regions that contribute to the scattering signal increases by ≈5% during the reaction at constant pressure, which is qualitatively in line with the observed macroscopic monolayer expansion. The chain tilt angle *φ* virtually does not change, but the chain cross-sectional area, *A*_0_ = *A*_*xy*_ cos(*φ*) increases significantly over time (see ESI†), indicating a less dense packing in the ordered regions after the reaction. In parallel, the Bragg peaks in the mid-angle region progressively disappear, indicating that the molecular superlattice gets destroyed during the reaction. Apparently, the hydrogen-bonding network is disrupted by the polymerization process. The systematically increasing width of the diffraction peaks of the chain lattice further indicates that the lateral order (correlation length, see ESI†) in the monolayers is getting drastically reduced upon cross-linking. Irrespective of the boundary condition regarding the trough area, the average crystalline domain size decreases from an initial value of *A*_d_ ≈ 800 nm^2^ down to *A*_d_ ≈ 50 nm^2^ within few hours of cross-linking time, see Fig. 4C. After 3 h of cross-linking time, all diffraction peaks have largely disappeared. The remaining broad intensity maximum resembles the ‘halo’ commonly observed in the WAXS-region in α-phases around 1.4 Å^−1^ indicating the short-range order of fluid chains.^30,31^ This observation results from the progress of the cross-linking reaction because the amines, which are part of the lipid hydrogen bond network, disappear due to the imine formation. It may be possible to control this expansion by the molecular dimensions of the cross-linker.

**Fig. 5:**
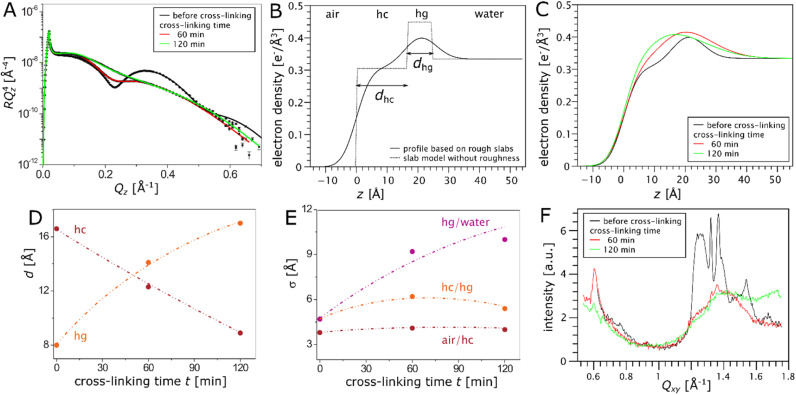
(A) XRR curves as *RQ*_*z*_^4^*vs. Q*_*z*_ from a TH4 monolayer on pH 10 subphase at π = 30 mN m^−1^ measured before and during the cross-linking reaction. Solid lines are the theoretical curves corresponding to the best-matching parameters in the two-slab-box model (panel (B)) of the interfacial electron density profile illustrated in panel C. (C) Reconstructed electron density profiles of the TH4 monolayer at π = 30 mN m^−1^ before and during the cross-linking reaction. (D) Thicknesses of the hydrocarbon (hc) and headgroup (hg) layers as functions of the cross-linking time. (E) Roughness of the interfaces between the different media as functions of the cross-linking time. Dash-dotted lines merely serve as guides to the eye. (F) Changes of the diffraction pattern induced by the cross-linking reaction as obtained in simultaneous GIXD measurements.

GIXD is the method of choice to monitor changes in the in-plane crystalline ordering of the monolayers during cross-linking reactions. In contrast, XRR, which provides information about the electron density profile of the monolayers perpendicular to the air–water interface, can be applied to both ordered and disordered layers and was used to monitor the cross-linking of the trivalent aminolipid TH4 in combination with GIXD. As seen in Fig. 1, this lipid has two alkyl chains of different lengths (C14 and C16) and 3 primary amine groups. It was intensively tested regarding its transfection efficiency,^32,33^ and cryo-transmission electron microscopy showed that this lipid is able to form ribbon and sheet structures which are interesting for material science.^34^ Strong intermolecular interactions in TH4 monolayers lead to a direct phase transition (re-sublimation) from a gaseous to an LC phase, in contrast to DiTT4, which exhibits a temperature-dependent first-order phase transition from a liquid-like LE phase to the LC phase.^22^ The presence of a Bragg peak at 1.325 Å^−1^ as seen in GIXD (Fig. 5F) is an indication of an H-bond network between the headgroups and resulting superlattice formation characterized by an additional peak ≈ 0.6 Å^−1^. Three intense Bragg peaks are associated with the chain lattice at all measured surface pressures, indicating that this lattice is oblique and does not change during lateral compression. The alkyl chains of TH4 are strongly tilted with respect to the surface normal.

The XRR curve of the TH4 monolayer on the pH 10 subphase at π = 30 mN m^−1^ before cross-linking (Fig. 5A) can be perfectly described by two homogeneous slabs for hydrocarbon chain (hc) and headgroup (hg) regions (Fig. 5B). The thicknesses *d* and electron densities *ρ* of the slabs, as well as their interfacial roughnesses *σ* are adjustable parameters (see tables in ESI†). Only the electron density of the subphase is fixed to the known value of water, *ρ*_w_ = 0.334 e^−^ Å^−3^. The hc layer has a comparatively low electron density, while the hg layer, due to the different composition, has a higher electron density. The thickness of the hc region, *d*_hc_ = 16.6 Å, is in excellent agreement with the theoretical thickness: *d* = *L* cos(32.6°) = 16.7 Å, with the chain tilt *φ* = 32.6° determined by GIXD (see Table S10 in the ESI†). The length of a chain in all-*trans* conformation is *L* ≈ (1.26*N* + 1.5) Å, with *N* as the number of CH_2_-groups per chain.^35,36^ The thickness of the hg region, *d*_hg_ = 8 Å, is plausible, too, when considering the molecular chemistry. The cross-linking reaction leads to drastic changes in the reflectivity curves (see Fig. 5A), which correspond to a progressive smearing out of the electron density profile with increasing layer roughness (Fig. 5C). The thickness of the hc region systematically decreases with the cross-linking time (Fig. 5D), which is in line with a fluidization of the chains and with the larger area requirement of the cross-linked headgroups seen in the GIXD data measured simultaneously (Fig. 5F), which will be discussed further below. The thickness of the hg layer, on the other hand, increases, which likely reflects changes in the headgroup orientation due to cross-linking and the effective addition of organic material in the form of the cross-linker. Fig. 5E shows the temporal evolution of the interfacial roughness. While the roughness of the air/hc interface is practically unchanged, the roughness between hg/water interface increases substantially over time due to the polymerization reaction. The roughness of the hc/hg interface does not seem to show a robust trend. The diffraction patterns are presented in Fig. 5F. The chain lattice has an oblique in-plane structure (*b* = 5.301 Å, *c* = 5.898 Å, *α* = 128.9°), and the two chains are strongly tilted (*φ* ≈ 33°, see tables ESI†). Additional Bragg peaks can be clearly seen, indicating the formation of a molecular superlattice. The supercell has an area of 146.0 Å^2^ (accommodating 3 TH4 molecules) and is commensurate with the hydrocarbon chain lattice (*b*′ = 2*b*, *c*′ = 3*c*). The most pronounced Bragg peaks at ≈0.6 Å^−1^ and 1.32 Å^−1^ correspond to peaks of the superlattice with Miller indices (−1 1), (1 −1) and (2 −1), (−2 1), respectively. After 120 min of reaction, a broad halo around 1.4 Å^−1^ (*A*_0_ = 23.3 Å^2^) starts dominating the diffraction pattern, indicating a transition from ordered to disordered chains. However, the peak at ≈0.6 Å^−1^ is still visible, either due to remaining ordered domains or due to a still existing superlattice with molten chains, which has never been reported to the best of our knowledge. For the moment, these scenarios cannot be finally discriminated and further investigations are needed.

Quite generally, the initial packing density of the monolayer may have a significant influence on the properties of the resulting sheets, an aspect that could be studied in the future. To this end, the experimental boundary condition with regard to constant pressure or constant area appears to be crucial. Keeping in mind that the lateral pressure in stiff or polymerized monolayers cannot be determined with high certainty using the Wilhelmy method, the most robust approach may be working at constant area while investigating the influence of the imposed area per lipid. The formation of macroscopically cross-linked nanosheets can then be assessed by electron microscopy, as demonstrated here.

## Experimental

### Chemicals

The lipids have been synthetized as previously reported with confirmed high purity (>95%).^37,38^ Chloroform and methanol were of the highest purity commercially available and purchased from Sigma-Aldrich (Merck KGaA, Germany). Milli-Q Millipore water with a specific resistance of 18.2 MΩ cm was used for all measurements and aqueous sample preparation. The cross-linker 1,3,5-triformylphoroglucinol (TFPG) (>98% purity) was purchased from Carbosynth Ltd. (Berkshire UK); and sodium hydroxide, sodium carbonate and sodium bicarbonate were purchased from Sigma-Aldrich (Merck KGaA, Germany).

### Sample preparation

The TFPG cross-linker solution was prepared by dissolving 10 mg of TFPG in an aqueous NaOH solution (0.07 M) followed by bath sonication for 2 h. The carbonate–bicarbonate buffer solution was prepared by mixing 27.5 mL of sodium carbonate solution (0.1 M) and 22.5 mL of sodium bicarbonate solution (0.1 M) and addition of deionized water to 200 mL. The pH value was adjusted to 9 or 10, respectively, by adding the appropriate amount of sodium hydroxide solution (1 M). The Langmuir trough was filled with the applied carbonate–bicarbonate buffer as subphase. Lipid solutions at a concentration of 1 mM in a chloroform : methanol 9 : 1 mixture (vol/vol) were spread onto the surface of the subphase. The paper plate Wilhelmy method was used to measure the surface pressure with an accuracy of ±0.1 mN m^−1^. Lateral compression to the desired pressure was started 15 min after the evaporation of the solvent with a compression rate below 10 Å^2^ per (molecule min). The TFPG cross-linker was injected from behind the barrier underneath the compressed monolayers (see ESI† for further details and limitations of this procedure). TFPG causes skin irritation [warning: skin corrosion/irritation]. NaOH and its solution, as well as the buffer at pH 10, can cause serious irritation of the skin and serious eye damage. It is mandatory to handle the solutions with protective gloves and eye protection. It is mandatory to handle chloroform under sufficient air exchange conditions. Isotherm measurements during the cross-linking process were not performed because partially cross-linked monolayers exhibit significant resistance to in-plane shear deformations, preventing reliable pressure measurements. Although isotherm measurements at a low cross-linking degree could be interesting in general, they would require very low cross-linker concentrations so that they can be recorded much faster than the progress of the reaction.

### IRRAS

The experiments were performed using an IFS 66 FTIR spectrometer equipped with a liquid-nitrogen-cooled mercury cadmium telluride detector attached to an external air/water reflection unit (XA-511, Bruker). In all experiments, the spectral resolution and scanner speed were 8 cm^−1^ and 20 kHz, respectively. The incident IR beam is polarized with a KRS-5 wire grid polarizer. Spectra are co-added over 200 scans for s-polarized light and over 400 scans for p-polarized light. A small reference trough and the larger sample trough are alternatively moved into the IR beam path by a shuttling mechanism. The principle of the method and its application to Langmuir films at the air/water interfaces have been described earlier.^39–41^ The measured signal represents the ratio of the reflected light from two liquid surfaces:RA = −log[(sample reflectivity)/(reference reflectivity)] = −log(*R*/*R*_0_)in reflectance/absorbance (RA) units. The two different light polarizations provide information on molecular orientation with respect to the surface plane of the monolayer. A change in the intensity ratio of p-polarized to s-polarized light (p/s ratio) for a vibrational band indicates a change in the average orientation of the vibration and thus of the molecule.

### TEM/SEM

The cross-linked Langmuir nanosheets were transferred to Quantifoil® Holey Carbon TEM grids as support (see ESI† for further details and limitations of this procedure). SEM images have been recorded on a Zeiss Merlin FE-SEM (Zeiss, Göttingen, Germany) equipped with a Gemini II™ column operating between 0.1 and 5 kV with a probe current between 60 and 190 pA. The (on axis) in-lens detector of the Gemini II column allowed for imaging at all magnifications. TEM images of the samples were recorded on a Philips CM12 electron microscope operating at 100 kV.

### X-ray diffraction and reflectometry

GIXD and XRR experiments were carried out either at the beamline ID10 of the European Synchrotron Radiation Facility (ESRF, Grenoble, France) or at the beamline P08 at storage ring PETRA III of Deutsches Elektronen Synchrotron (DESY, Hamburg, Germany). At ID10, the incident beam had an energy of 10 keV energy, corresponding to a wavelength of *λ* = 1.239 Å. At P08, the beam energy was 15 keV (*λ* = 0.826 Å). In both cases, the Langmuir trough was enclosed in a helium-filled sealed container with Kapton windows for the X-ray beam. The subphase temperature was maintained at 20 °C with a water flow thermostat, and constantly monitored.

### GIXD (ID10 and P08)

The incident beam strikes the air/water interface at a grazing angle of *θ*_i_ ≈ 0.8*θ*_cr_ with *θ*_cr_ being the critical angle of total reflection for water, such that only the immediate vicinity of the interface is probed with an evanescent wave with a decay length of ≈8 nm. The beam footprint on the water surface was 0.12 mm × 35 mm (ID10) or 1 mm × 60 mm (P08). The diffraction signal was collected with 1D linear detector (ID10: Mythen2 2K; P08: Mythen2 1K; PSI, Villigen, Switzerland) by scanning the azimuth angle *Δ* and, with that, the in-plane component *Q*_*xy*_ = (4π/*λ*)sin(*Δ*/2) of the scattering vector *Q* = (*Q*_*xy*_, *Q*_*z*_)^*T*^. The out-of-plane component, *Q*_*z*_ = (2π/*λ*)[sin(*θ*) + sin(*θ*_i_)], is encoded in the vertical position of the detector channels, where *θ* denotes the angle between the scattered beam and the sample plane. The in-plane beam divergence was collimated with a Soller collimator placed in front of the detector providing at ID10 δ*Δ* ≈ 0.08° (FWHM), corresponding to *w*^res^_*xy*_ = (4π/*λ*)sin(δ*Δ*/2) = 0.007 Å^−1^, and at P08 δ*Δ* ≈ 0.09°, corresponding to *w*^res^_*xy*_ = 0.012 Å^−1^. To reconstruct the 2D-crystalline structure of the monolayers, the diffraction peaks on the resulting intensity maps *I*(*Q*_*xy*_, *Q*_*z*_) were analysed as described previously.^42,43^ In brief, the *Q*_*xy*_- and *Q*_*z*_-positions and widths of the peaks were obtained in a least-squares fitting procedure, and the lattice and superlattice parameters (unit cell dimensions, in-plane area per chain *A*_*xy*_, cross-sectional area per chain *A*_0_, chain tilt angle *φ*) were then reconstructed from the peak positions as described earlier.^35,43,44^ Finally, the average areas of the ordered domains were obtained from the peak widths as detailed in ref. 43.

### XRR (only ID10)

Reflectivity curves were measured in the *θ*–*θ* geometry. The detector was a Mythen2 1K (PSI, Villigen, Switzerland), collimation is provided by the pixels (50 μm vertical, 8 mm horizontal), which act as detector slits. Undesired scattering background was minimized with a pair of post-sample slits with 1 mm horizontal gap and 2 mm vertical gap, placed 0.5 m before the detector. The reflectivity *R* is defined as the ratio between reflected and incident intensities. The measured angular reflectivity curves, *R*(*θ*), were transformed to *Q*_*z*_-dependent reflectivity curves *R*(*Q*_*z*_) according to the relation *Q*_*z*_ = (4π/*λ*)sin *θ*. For analysis, the experimental data were compared with theoretically modelled XRR curves based on a slab-model representation of the electron density profiles of the interfacial lipid layers (see Results and discussion section), as described in an earlier work.^45^ These profiles were discretized into 1 Å-thin sub-slabs of constant electron density, and the corresponding *Q*_*z*_-dependent reflectivities were then calculated from the Fresnel reflection laws at each slab–slab interface using the iterative recipe of Parratt.^46^ Finally, all model parameters (electron densities, layer thicknesses, and roughness) were varied until the best agreement with the experimental data was reached *via χ*^2^ minimization.

## Conclusions

We have investigated the process of cross-linking reactions in aminolipid monolayers at air/water interfaces with various surface-sensitive techniques as proof-of-concept study. Resource-related limitations restricted the application of all methods to all lipids, but nevertheless the informative value of each single method was demonstrated. While IRRAS allows following the formation of functional groups through cross-linking, GIXD and XRR can be used to monitor the cross-linking process in terms of structural changes along the surface plane and perpendicular to it, respectively. Initially, the used aminolipids were synthesized as new transfection agents with different protonable headgroups and different well-defined area requirements of hydrophobic and hydrophilic groups and therefore different packing densities. Here, their films were polymerized by the efficient and well-established imine condensation reaction, which does not require expensive catalysts and enables film annealing due to its reversibility and thus the creation of highly ordered crystalline materials.^16^ This method also introduces imine bonds (–HC=N–) into the structure, which can promote metal ion (M^*x*+^) and CO_2_ binding, which may be interesting for future use as biomineralization template or as thin films in electronics. We demonstrated that this reaction is applicable to lipid monolayers at the soft air/water interface, resulting in Janus-faced 2D nanosheets with one hydrophobic face and one functionalizable hydrophilic face that may be used for various purposes in the fields of soft matter and biomimetic materials. Starting with highly ordered condensed monolayers, the polymerization reaction for both lipid species studied with X-rays resulted in monolayers with less ordered chains (liquid-ordered) but strongly cross-linked headgroups. The TFPG-based reaction was applicable to lipids with comparable head group structure but different packing parameters (TH4, DiTT4), and also to lipids with a different head group structure (TH14).

## Conflicts of interest

There are no conflicts to declare.

## Supplementary Material

NA-005-D3NA00244F-s001
