# Supplementary material for: Cross-linking reactions in Langmuir monolayers of specially designed aminolipids – a toolbox for the customized production of amphiphilic nanosheets
Source: Nanoscale Adv. 2023 Aug 9;5(17):4589–97. doi: 10.1039/d3na00244f (PMC10448339; doi:10.1039/d3na00244f)
Supplement: NA-005-D3NA00244F-s001 [file NA-005-D3NA00244F-s001.pdf]

Electronic Supplementary Material (ESI) for Nanoscale Advances.
This journal is © The Royal Society of Chemistry 2023

Cross-linking Reactions in Langmuir Monolayers of Specially Designed Aminolipids – A Toolbox for the Customized Production of Amphiphilic Nanosheets

Cristina Stefaniu,[a] Christian Wölk,[b,*] Victoria M. Latza,[a] Andrei Chumakov,[c] Gerald Brezesinski [a,d] and Emanuel Schneck,[a,d,*]

[a] *Departments of Biomaterials and Biomolecular Systems, Max Planck Institute of Colloids and Interfaces, Am Mühlenberg 1, 14476 Potsdam, Germany.*
[b] *Pharmaceutical Technology, Faculty of Medicine, University of Leipzig, Eilenburger Str. 15a, 04317 Leipzig, Germany. E-mail: christian.woelk@medizin.uni-leipzig.de*
[c] *European Synchrotron Radiation Facility, 71, avenue des Martyrs, CS 40220, 38043 Grenoble Cedex 9, France.*
[d] *Department of Physics, TU Darmstadt, Hochschulstr. 8, 64289 Darmstadt, Germany. E-mail: emanuel.schneck@pkm.tu-darmstadt.de*

Details and limitations of the cross-linker injection underneath the pre-arranged monolayer

The TFPG cross-linker was injected from behind the barrier underneath the compressed monolayers by using a regular syringe with a 15 cm long needle. The needle was bent by 90-degree relatively close to the tip of the plastic syringe. During the injection, the syringe remained behind the barriers and the tip of the needle was systematically moved to virtually all regions in order to minimize cross-linker concentration gradients in the subphase. While some concentration gradients will nevertheless inevitably occur, which can result in a laterally heterogeneous reaction speed, the influence on the results of the employed surface-sensitive techniques (IRRAS, XRR, GIXD) can be considered minor, because no pronounced concentration gradients are expected to occur underneath their measurement footprints.

Details and limitations of the transfer of cross-linked nanosheets onto EM grids

The cross-linked Langmuir nanosheets were transferred to Quantifoil® holey carbon TEM grids as support by simply picking up the film with tweezers from different regions. During this procedure, bending of the grid and its lateral displacement within the interface can lead to the formation of wrinkles. After allowing the sample to slowly dry in a gentle nitrogen stream, the covered TEM grids were directly used for imaging.

Limitations of the pressure sensing with a Wilhelmy plate

Pressure sensing with the Wilhelmy technique is not well suited for stiff monolayers such as highly crystalline or polymerized films. The reason is that stiff monolayers in combination with moving barriers can push the Wilhelmy plate to the side, resulting in a tilt of the plate and, in turn, leading to possible deviations in the recorded pressure value. Pressures recorded during cross-linking must therefore be considered apparent values with limited accuracy. They were therefore not interpreted on a quantitative level in this study.

DiTT4 monolayers on pH 10 measured at constant pressure (30 mN/m)

Cross-linking reaction time [min]	*π [mN/m]*	*Q_{xy1} FWHM [Å^{-1}]*	*Q_{z1} FWHM [Å^{-1}]*	*Q_{xy2} FWHM [Å^{-1}]*	*Q_{z2} FWHM [Å^{-1}]*	*Q_{xy3} FWHM [Å^{-1}]*	*Q_{z3} FWHM [Å^{-1}]*
0	30	1.391<br>0.020	0.092<br>0.34	1.496<br>0.022	0.495<br>0.34	1.534<br>0.024	0.403<br>0.34
60	30	1.362<br>0.027	0.132<br>0.34	1.472<br>0.027	0.525<br>0.34	1.506<br>0.038	0.393<br>0.34
120	30	1.372<br>0.054	0.128<br>0.34	1.456<br>0.071	0.518<br>0.34	1.504<br>0.096	0.390<br>0.34
180	30	1.378<br>0.086	0.139<br>0.34	1.467<br>0.066	0.479<br>0.34	1.506<br>0.075	0.340<br>0.34

Table S1. GIXD results of DiTT4 monolayers on pH 10 measured at constant pressure (30 mN/m) and 20 °C before and after 60, 120, and 180 min reaction time: Bragg peak Q_{xy} (± 0.003 Å^{-1}) and rod Q_z (± 0.005 Å^{-1}) positions and their corresponding full-width at half-maximum (*FWHM(xy)* ± 0.003 Å^{-1} and *FWHM(z)* ± 0.01 Å^{-1}) are presented.

Cross-linking reaction time [min]	*π [mN/m]*	*a/b/c [Å]*	*α/β/γ [°]*	*distortion d*	*tilt φ [°]*	*A_{xy} [Å^2]*	*A_0 [Å^2]*
0	30	4.670<br>5.023<br>5.150	125.4<br>118.7<br>115.9	0.1147	18.9	21.1	20.0
60	30	4.746<br>5.130<br>5.248	125.6<br>118.5<br>115.9	0.1185	19.9	21.9	20.6
120	30	4.794<br>5.088<br>5.255	124.8<br>119.4<br>115.8	0.1061	19.9	22.0	20.6
180	30	4.772<br>5.080<br>5.215	124.8<br>119.0<br>116.2	0.1035	18.3	21.8	20.7

Table S2. GIXD results of DiTT4 monolayers on pH 10 measured at constant pressure (30 mN/m) and 20 °C: Lattice parameters *a*, *b*, *c* and *α*, *β*, *γ*, lattice distortion (*d*), chain tilt (*φ*) from the surface normal, in-plane area per alkyl chain (A_{xy}) and chain cross-sectional area (A_0).

Q_{xy} *cal* [Å^{-1}]	Q_{xy} *exp* [Å^{-1}]	(h_s k_s)	a^s, b^s, γ^s [Å, Å, °]	A^{crys} [Å^2]
0.998	0.992	(0 -2), (0 2)	10.046, 15.450, 125.4	126.5
1.132	1.130	(1 1), (-1 -1)		
1.224	1.225	(1 -3), (-1 3)		
1.256	1.254	(2 -2), (-2 2)		
1.310	1.312	(2 -1), (-2 1)		
1.391	1.391	(2 -3), (-2 3)		
1.497	1.496	(0 -3), (0 3)		
1.535	1.534	(2 0), (-2 0)		
1.572	1.576	(1 2, (-1 -2)		
1.670	1.672	(-2 4), (2 -4)		
1.673		(-1 4), (1 -4)		

Table S3. Experimental and calculated Q_{xy} data for a DiTT4 monolayer on a pH 10 subphase at 30 mN/m based on a superlattice with lattice parameters a^s, b^s, γ^s. (h_s k_s) are the Miller indices, A^{crys} (± 0.5 Å^2) is the in-plane area of the corresponding superlattice.

Cross-linking reaction time [min]	$L_{xy}(1)/L_{xy}(2)/L_{xy}(3)$ [Å]	A_d [nm^2]
0	353<br>314<br>269	822
60	236<br>226<br>157	321
120	107<br>81<br>60	56
180	67<br>87<br>76	50

Table S4. Average sizes A_d of ordered domains for DiTT4 monolayers at π = 30 mM/m on pH 10 measured at 20 °C before and after 60, 120, and 180 min reaction time. The in-plane correlation lengths associated with the three peaks of the chain lattice $L_{xy}(i)$ are computed according to the Scherrer equation.

DiTT4 monolayers on pH 10 measured at constant area (starting pressure - 30 mN/m)

Cross-linking reaction time [min]	*π [mN/m]*	*Q_{xy1} FWHM [Å^{-1}]*	*Q_{z1} FWHM [Å^{-1}]*	*Q_{xy2} FWHM [Å^{-1}]*	*Q_{z2} FWHM [Å^{-1}]*	*Q_{xy3} FWHM [Å^{-1}]*	*Q_{z3} FWHM [Å^{-1}]*
0	30	1.388<br>0.018	0.073<br>0.34	1.493<br>0.019	0.475<br>0.34	1.529<br>0.019	0.402<br>0.34
30	34.3	1.397<br>0.029	0.065<br>0.34	1.493<br>0.031	0.462<br>0.34	1.523<br>0.025	0.397<br>0.34
60	34.5	1.396<br>0.035	0.065<br>0.34	1.491<br>0.053	0.422<br>0.34	1.525<br>0.053	0.357<br>0.34
120	38.6	1.382<br>0.085	0.088<br>0.34	1.476<br>0.076	0.423<br>0.34	1.494<br>0.085	0.335<br>0.34

Table S5. GIXD results of DiTT4 monolayers on pH 10 measured at constant area (starting pressure - 30 mN/m) and 20 °C before and after 30, 60, and 120 min reaction time: Bragg peak Q_{xy} (± 0.003 Å^{-1}) and rod Q_z (± 0.005 Å^{-1}) positions and their corresponding full-width at half-maximum (*FWHM(xy)* ± 0.003 Å^{-1} and *FWHM(z)* ± 0.01 Å^{-1}) are presented.

Cross-linking reaction time [min]	*π [mN/m]*	*a/b/c [Å]*	*α/β/γ [°]*	*distortion d*	*tilt φ [°]*	*A_{xy} [Å^2]*	*A_0 [Å^2]*
0	30	4.683<br>5.037<br>5.159	125.3<br>118.7<br>116.0	0.1137	18.4	21.2	20.1
30	34.3	4.702<br>5.026<br>5.127	124.8<br>118.7<br>116.5	0.1022	18.1	21.1	20.1
60	34.5	4.702<br>5.022<br>5.136	124.9<br>118.8<br>116.3	0.1039	16.5	21.2	20.3
120	38.6	4.781<br>5.106<br>5.168	124.5<br>118.4<br>117.1	0.0946	16.5	21.7	20.8

Table S6. GIXD results of DiTT4 monolayers on pH 10 measured at constant area (starting pressure - 30 mN/m) and 20 °C before and after 30, 60, and 120 min reaction time: Lattice parameters *a*, *b*, *c* and *α*, *β*, *γ*, lattice distortion (*d*), chain tilt (*φ*) from the surface normal, in-plane area per alkyl chain (A_{xy}) and chain cross-sectional area (A_0).

Cross-linking reaction time [min]	*$L_{xy}(1)/L_{xy}(2)/L_{xy}(3)$ [Å]*	*A_d [nm^2]*
0	435<br>377<br>377	1420
30	217<br>195<br>257	434
60	171<br>109<br>109	161
120	67<br>75<br>67	44

Table S7. Average sizes A_d of ordered domains for DiTT4 monolayers on pH 10 measured at constant area (starting pressure - 30 mN/m) and 20 °C before and after 30, 60, and 120 min reaction time. The in-plane correlation lengths associated with the three peaks of the chain lattice $L_{xy}(i)$ are computed according to the Scherrer equation.

TH4 monolayers on pH 10 measured at constant area (starting pressure - 30 mN/m)

	d (Å)			*ρ* ($e^-/Å^3$)			*σ* (Å)		
time (min)	0	60	120	0	60	120	0	60	120
hc	16.6	12.3	8.9	0.305	0.304	0.327	3.8	4.1	4.0
hg	8.0	14.1	17.0	0.449	0.464	0.433	4.6	6.2	5.4
hg/water							4.7	9.1	10.2

Table S8. Parameters *d* (thickness), *ρ* (electron density), and *σ* (roughness) of the best-matching 2-box model for a TH4 monolayer at constant area. The labels 'hc' and 'hg' stand for monolayer tails and monolayer headgroups, respectively. The reaction started at π = 30 mN/m (time 0). The XRR has been also measured after 60 and 120 min of reaction.

Cross-linking reaction time [min]	*π [mN/m]*	Q_{xy1} *FWHM [Å⁻¹]*	Q_{z1} *FWHM [Å⁻¹]*	Q_{xy2} *FWHM [Å⁻¹]*	Q_{z2} *FWHM [Å⁻¹]*	Q_{xy3} *FWHM [Å⁻¹]*	Q_{z3} *FWHM [Å⁻¹]*
0	30	1.255<br>0.076	0.784<br>0.33	1.369<br>0.038	0.461<br>0.33	1.523<br>0.109	0.323<br>0.33
30	30.8	1.244<br>0.063	0.820<br>0.33	1.352<br>0.051	0.455<br>0.33	1.505<br>0.125	0.365<br>0.33
60	32	1.237<br>0.090	0.796<br>0.33	1.355<br>0.093	0.432<br>0.33	1.492<br>0.133	0.364<br>0.33
120	33.1			1.402<br>0.255			

Table S9. GIXD results of TH4 monolayers on pH 10 measured at constant area (starting pressure - 30 mN/m) and 20 °C before and after 30, 60, and 120 min reaction time: Bragg peak Q_{xy} (± 0.003 Å^{-1}) and rod Q_z (± 0.005 Å^{-1}) positions and their corresponding full-width at half-maximum (*FWHM(xy)* ± 0.003 Å^{-1} and *FWHM(z)* ± 0.01 Å^{-1}) are presented.

Cross-linking reaction time [min]	*π [mN/m]*	*a/b/c [Å]*	*α/β/γ [°]*	*distortion d*	*tilt φ [°]*	A_{xy} *[Å²]*	A_0 *[Å²]*
0	30	4.860<br>5.301<br>5.898	128.9<br>121.9<br>109.2	0.2250	32.6	24.3	20.5
30	30.8	4.923<br>5.351<br>5.956	128.7<br>122.0<br>109.3	0.2221	33.9	24.9	20.6
60	32	4.931<br>5.401<br>5.947	128.8<br>121.3<br>109.9	0.2161	33.1	25.0	21.0

Table S10. GIXD results of TH4 monolayers on pH 10 measured at constant area (starting pressure - 30 mN/m) and 20 °C before and after 30, 60, and 120 min reaction time: Lattice parameters *a*, *b*, *c* and *α*, *β*, *γ*, lattice distortion (*d*), chain tilt (*φ*) from the surface normal, in-plane area per alkyl chain (A_{xy}) and chain cross-sectional area (A_0).
